# Stretchable supramolecular hydrogels with triple shape memory effect†
†Electronic supplementary information (ESI) available. See DOI: 10.1039/c6sc02354a



**DOI:** 10.1039/c6sc02354a

**Published:** 2016-07-07

**Authors:** Xiaoxia Le, Wei Lu, Jing Zheng, Dingyi Tong, Ning Zhao, Chunxin Ma, He Xiao, Jiawei Zhang, Youju Huang, Tao Chen

**Affiliations:** a Division of Polymer and Composite Materials , Ningbo Institute of Material Technology and Engineering , Chinese Academy of Science , Ningbo , 315201 , China . Email: zhangjiawei@nimte.ac.cn ; Email: tao.chen@nimte.ac.cn; b Beijing National Laboratory for Molecular Sciences , Laboratory of Polymer Physics and Chemistry , Institute of Chemistry , Chinese Academy of Sciences , Beijing , 100190 , China

## Abstract

Shape memory polymers based on reversible supramolecular interactions have invoked growing research interest, but still suffer from limitations such as poor mechanical strength and finite shape memory performance. Here, we present a novel mechanical stretchable supramolecular hydrogel with a triple shape memory effect at the macro/micro scale. The introduction of a double network concept into supramolecular shape memory hydrogels endows them with excellent mechanical properties. The design of two non-interfering supramolecular interaction systems of both dynamic phenylboronic (PBA)–diol ester bonds and the chelation of alginate with Ca^2+^ endues the hydrogel with outstanding triple shape memory functionalities.

## Abstract 2

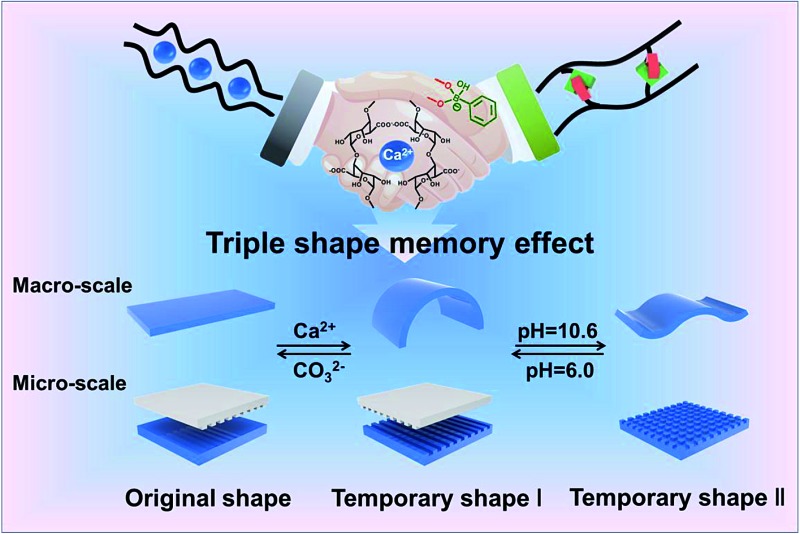
Here, we present a novel mechanical stretchable supramolecular hydrogel with a triple shape memory effect at the macro/micro scale.

## Introduction

As one of the most important types of stimuli-responsive polymers, shape memory polymers (SMPs) are able to return to their original shapes from preprogrammed temporary shapes upon a trigger *via* external stimuli,^1–8^ they thus have attracted tremendous attention and shown promising applications in many fields such as biomedical, textile, aerospace and so on.^9–11^ Early SMPs can only remember one temporary shape in each shape memory cycle, resulting in a dual shape memory effect. Since the potential application of SMPs is determined normally by the number of temporary shapes that can be fixed, increasing attention has thus been paid to constructing multi-SMPs, which could stabilize two or more temporary shapes.^12–16^ Most of the current SMPs are made from thermo-responsive polymers, in which temporary shapes are fixed by vitrification or crystallization of switching domains, and the shape recovery is induced by heat.^17^ As heat is not a convenient stimulus in practical biomedical and textile applications, other external stimuli such as light, electricity, magnetic field and chemicals have become increasingly attractive because of their corresponding shape memory ability at ambient temperatures.^18–23^

Due to the reversible and dynamic nature of supramolecular interactions,^24–26^ there is a great advantage in introducing them into SMPs. Some of the reversible switches such as metal–ligand binding, host–guest interactions and dynamic covalent bonds *etc.* have recently been used as temporary crosslinks to realize shape memory performance.^27–32^ Rowan *et al.* ^27^ reported a first example to demonstrate the use of metal–ligand crosslinks for accomplishing a shape memory effect. Liu^28^ and Yang^29^ utilized Zn^2+^–imidazole and Fe^3+^–phosphate coordination respectively to construct hydrogels with shape memory behavior. Zhang^31^ and Harada^32^ recently showed that host–guest complexes between dextrin and guest molecules such as adamantine and ferrocene can also be applied to fix temporary shapes. These pioneering works are tremendously useful to inspire the design of novel SMPs on the basis of supramolecular interactions. In our previous work, we have also successfully applied alginate–Ca^2+^ coordination to achieve a shape memory hydrogel.^33^ Although constructing SMPs on the basis of supramolecular switches is an efficient way to realize shape deformation and recovery independent of heat, there are still some challenges to develop high mechanical strength, and multi-shape memory supramolecular hydrogels for real potential applications.

It is well known that double network (DN) structures could be used to improve the mechanical strength of hydrogels.^34,35^ The DN concept in which a supramolecular network and a chemical crosslinked network are introduced into supramolecular shape memory hydrogels renders them with ultra-stretchability. The supramolecular network acts as a “switching segment” to fix the temporary shape, while the chemical crosslinked network serves as a “hard segment” to memorize the original shape. In addition, the design of two non-interfering supramolecular interaction systems of both dynamic PBA–diol ester bonds and the chelation of alginate with Ca^2+^ endues the hydrogel with a triple shape memory effect.

**Fig. 1:**
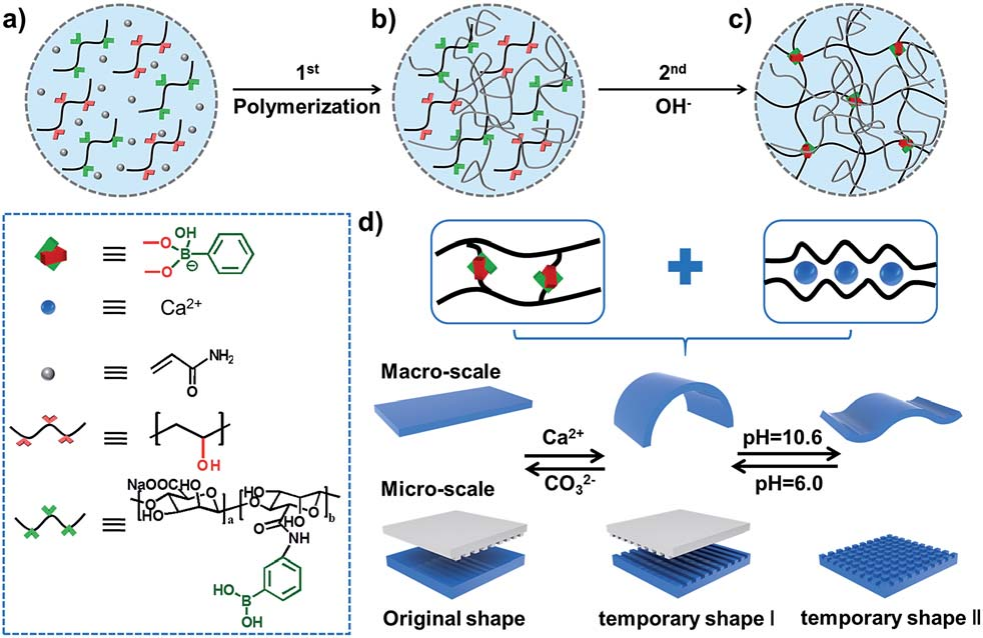
Schematic illustration of stretchable supramolecular hydrogels with a triple shape memory effect. (a–c) Acrylamide is polymerized in the presence of Alg-PBA and PVA, then the supramolecular network is formed *via* immersion into alkaline solution to generate dynamic PBA–diol ester bonds. (d) The reversible PBA–diol ester bonds and the chelation of Alg/Ca^2+^ endow the hydrogel with triple shape memory behavior at both the macro-scale and micro-scale.

The preparation process for stretchable supramolecular hydrogels with a triple shape memory effect is illustrated in Fig. 1. Acrylamide (AAm) was polymerized firstly in the presence of phenylboronic acid grafted alginate (Alg-PBA) and poly(vinyl alcohol) (PVA) (Scheme S1 and Fig. S1†). After immersion in alkaline solution to generate reversible PBA–diol ester bonds (Fig. S2†), hydrogels with good mechanical performance due to their DN structure are obtained (Fig. 1c). Di- and trivalent cations such as Ca^2+^ can chelate with the alginate and stabilize the temporary shape (Fig. S2†), moreover, the reversible PBA–diol ester bonds could also be applied to memorize the temporary shape and render the hydrogel shape memory property (Fig. 1d). Therefore, a triple shape memory effect is achieved and stepwise shape fixing and shape recovery can be realized *via* programmable changes in the external stimuli. To the best of our knowledge, our approach is the first investigation to endow SMPs with a triple shape memory effect at both the macro- and the micro-scale on the basis of dual non-interfering supramolecular interactions, which will broaden the list of SMPs and inspire the design and fabrication of novel supramolecular SMPs.

## Results and discussion

**Fig. 2:**
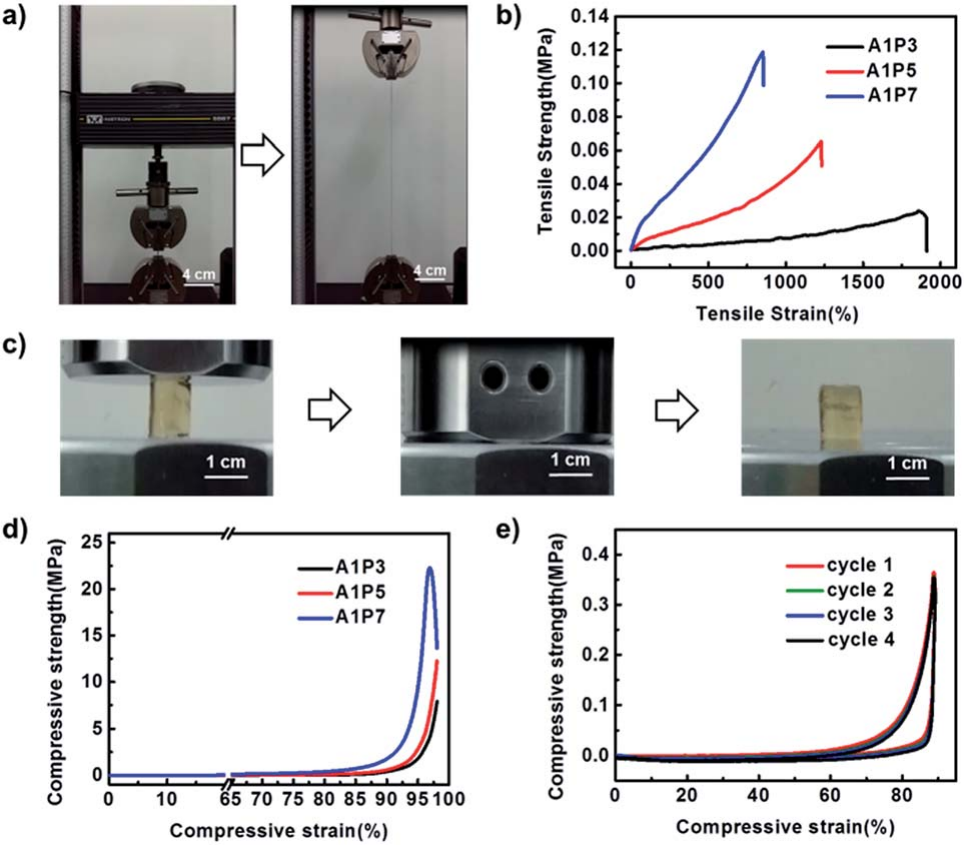
The mechanical properties of the as-prepared DN supramolecular hydrogels. (a) Tensile test, measured at an extension rate of 50 mm min^–1^ and a temperature of 25 °C. (b) Tensile stress–strain curves of DN hydrogels with different network ratios. (c) Compression test, measured at a compression rate of 10% original height per min and the final compressive strain is 98%. (d) Compressive stress–strain curves of the DN hydrogels. (e) The cyclic compression of A1P5, measured at a compression rate of 10% original height per min, the maximum compressive deformation is 90%.

Unlike other shape memory supramolecular hydrogels, our DN hydrogels display excellent mechanical properties because the supramolecular network serves as a sacrificial network, which can dissipate stress generated during the deformation. As shown in Fig. 2a, S4 and Movie S1,† the hydrogel can be stretched to more than 12 times its size and recover to its initial state. In order to investigate the influence of the ratio of the two networks on the mechanical performance of the hydrogel, three hydrogels with the ratios of supramolecular network to polyacrylamide network as 1 : 3 (A1P3), 1 : 5 (A1P5) and 1 : 7 (A1P7) are prepared (Table S1†). As shown in Fig. 2b and S5,† with the increment of the polyacrylamide network, the tensile stress at break increases from 0.03 MPa to 0.12 MPa, while the elongation at break decreases from 1800% to 850%. In addition, the compressive strength also increases with the increasing ratio of polyacrylamide network (Fig. 2d, S6, Movie S3†). As the A1P5 hydrogel exhibits good mechanical performance (Fig. 2c), and it is tough enough to withstand four consecutive cyclic compressions without obvious damage (Fig. 2e), it was therefore chosen for the following investigation.

**Fig. 3:**
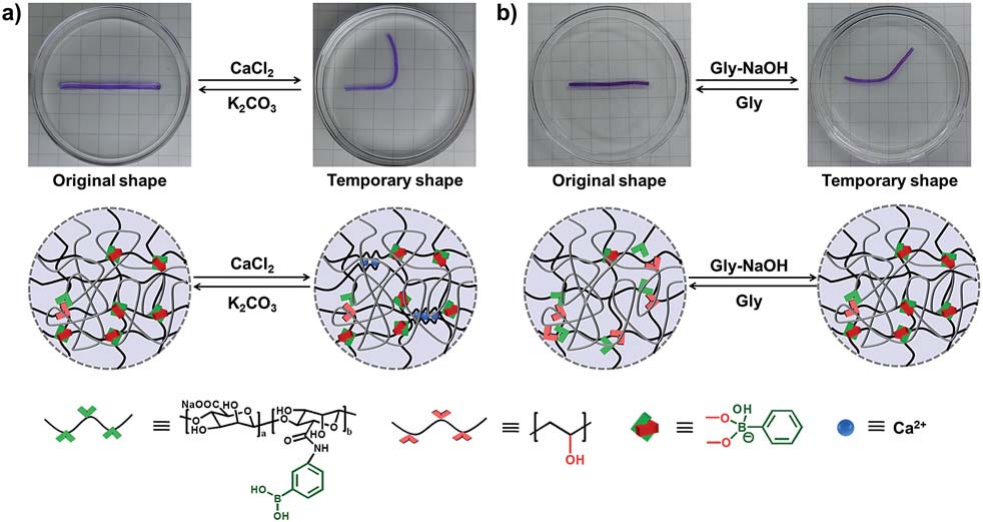
Dual shape memory effect at the macro-scale. (a) Shape memory behaviour based on Alg/Ca^2+^: the deformed hydrogel can fix its shape in CaCl_2_ solution through the chelation between alginate and Ca^2+^. Ca^2+^ can be erased by CO_3_^2–^ leading to the shape recovery. (b) Shape memory behaviour based on PBA–diol ester bonds: the deformed hydrogel can form PBA–diol ester bonds in Gly–NaOH aqueous solution (pH = 10.6), thus fixing its temporary shape, and can recover to its original shape *via* immersion in Gly solution (pH = 6) to break the temporary PBA–diol crosslinks.

As we all know, the α-l-guluronate (G unit) of the alginate chain can chelate with Ca^2+^ to form an egg-box-like structure,^36–39^ and the interactions between alginate and Ca^2+^ can be applied as “temporary crosslinks” to stabilize the temporary shape, and render the DN hydrogel with shape memory properties. As shown in Fig. 3a, a straight strip of supramolecular hydrogel is deformed into a “U” shape and immersed into CaCl_2_ solution (0.1 M), and the temporary shape can be fixed in 1 min because of the formation of Alg–Ca^2+^ crosslinks. After immersion into K_2_CO_3_ solution, Ca^2+^ will be extracted by CO_3_^2–^, the resultant hydrogel will slowly recover to its original shape (Fig. S7†), and the shape recovery process can be cycled at least 4 times (Fig. S8†). In addition, the non-interfering dynamic PBA–diol ester bonds can also be employed to fix the temporary shape of the hydrogel (Fig. 3b). When a straight strip of hydrogel was immersed into glycine aqueous solution (pH = 6) for 8 min to destroy most of the PBA–diol ester bonds, the hydrogel became soft and could be deformed. The bent hydrogel is then transferred into glycine–NaOH aqueous solution (pH = 10.6) to induce the formation of PBA–diol ester bonds, which resulted in the fixing of the deformed shape. When the hydrogel with a fixed temporary shape is immersed into glycine aqueous solution (pH = 6), the disassociation of the dynamic PBA–diol ester bonds will lead to the recovery of the original shape (Fig. S9†).

**Fig. 4:**
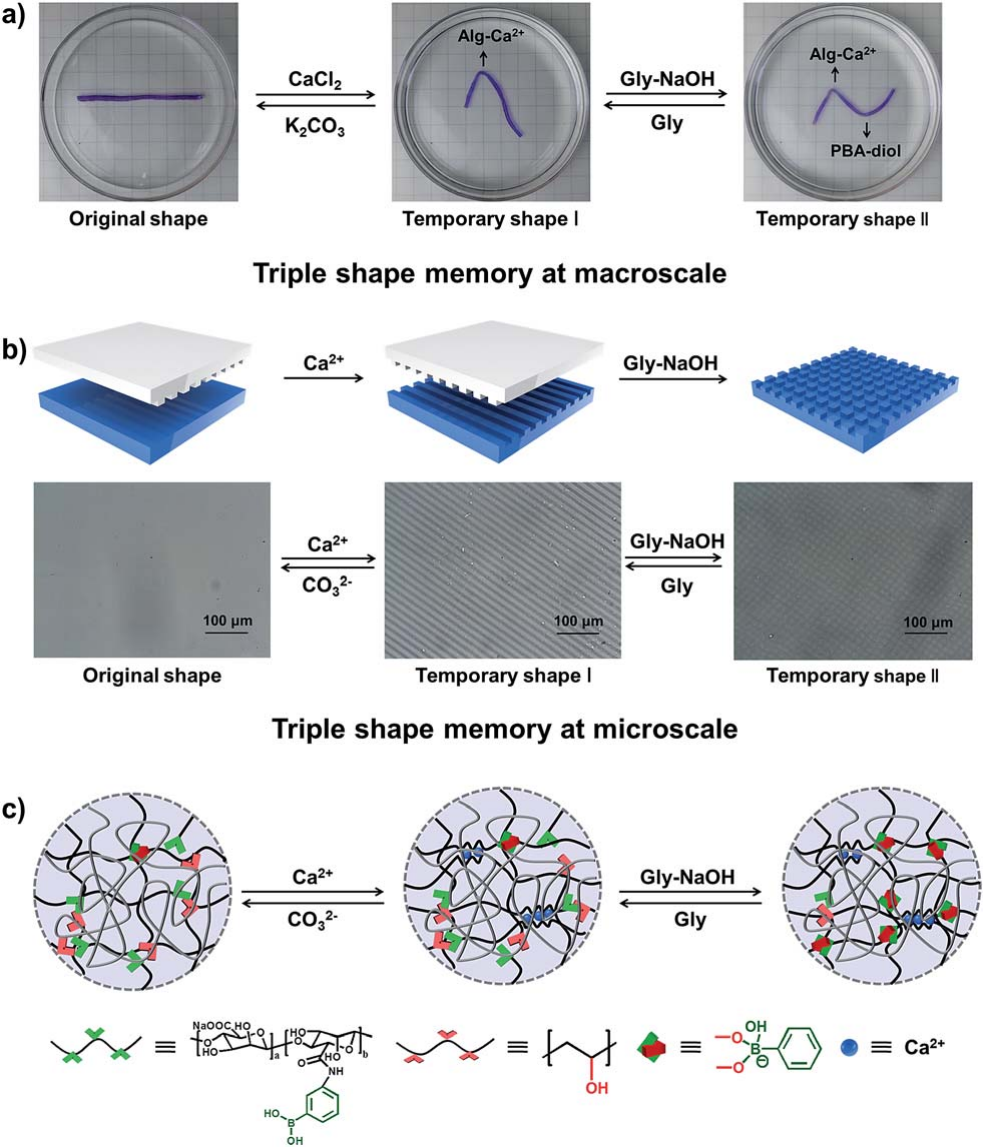
Triple shape memory effects. (a) Triple shape memory at the macro-scale. (b) Triple shape memory behaviour at the micro-scale, microscope images of original hydrogel, band-memorized hydrogel and lattice-memorized hydrogel. (c) The mechanism of the triple shape memory effect: the original hydrogel can fix its temporary shape I in CaCl_2_ solution and memorize temporary shape II in Gly–NaOH solution (pH = 10.6). By immersion in Gly solution (pH = 6) and K_2_CO_3_ solution step by step, the hydrogel can recover to the original shape.

Currently known materials with triple shape memory effects are capable of maintaining two temporary conformations by two discrete thermal transitions or molecular switches.^13,40^ As our supramolecular hydrogels can respond to two independent stimuli, it is envisioned that they have triple shape memory abilities. As a simple example shown in Fig. 4a, a straight strip of hydrogel is first deformed and fixed in CaCl_2_ solution to get temporary shape I, then it can be deformed again and immersed into glycine–NaOH aqueous solution (pH = 10.6) to stabilize temporary shape II. The bent hydrogel with “N” shape will recover from temporary shape II to temporary shape I by breaking the PBA–diol ester bonds. It will finally become straight because of the disassociation of the Alg–Ca^2+^ crosslinks upon immersion into K_2_CO_3_ solution (Fig. S10†). Moreover, the programmable stretching shape memory and releasing shape recovery were also investigated (Fig. S11†). The strategy introduced here selects two different kinds of supramolecular interactions without interference, making it possible to realize triple shape memory properties on the basis of supramolecular interactions.

Although the microscopic shape memory performance has been extensively investigated, the shape memory ability at the micro-scale is highly desired because of the potential applications in micro-optical devices, and remains a challenge due to the inconvenience of precisely controlling the micro-features.^41–43^ The outstanding triple shape memory ability of the as-prepared hydrogel at the macro-scale makes it possible to deform and recover surface features at the micro-scale. Our strategy for achieving a micro-patterned surface is schematically shown in Fig. 4b. A structured polydimethylsiloxane (PDMS) stamp is first brought into contact with a thin layer of hydrogel with gentle force to create micro-patterns in line onto the surface *via* a micro-contact printing method (μCP). The hydrogel is then soaked in CaCl_2_ solution for 1 min before removing the PDMS stamp to fix the line patterns by the Alg–Ca^2+^ complexation. The PDMS stamp is subsequently turned 90 degrees and brought into contact with the hydrogel again to create further micro-patterns in grid. The hydrogel is finally immersed into Gly–NaOH (pH = 10.6) solution to stabilize the second temporary shape through the formation of dynamic PBA–diol ester bonds. Fig. 4b shows the optical microscope image of the resulting hydrogel with line or grid micro-patterns in size of about 10 μm, which is in good agreement with that of the PDMS stamp. By breaking the dynamic PBA–diol ester bonds and Alg–Ca^2+^ coordination, the micro-patterns on the surface of the hydrogel can be erased step by step. As shown in Fig. S12,† after immersion into Gly aqueous solution to break the PBA–diol ester bonds, the hydrogel with line patterns in the shape recovery process is almost identical to the middle images of Fig. 4b, which suggests the shape recovery ratio is almost 100%. As far as we are aware, this is the first example to accomplish triple shape memory effect at the micro-scale by taking advantage of two kinds of supramolecular interactions, which will expand the potential applications of supramolecular interaction induced SMPs.

The self-healing behaviour is a general phenomenon in nature, in which most organisms have the ability to self-heal upon encountering damages. It is highly important for the healed organisms to retain not only the structure, but also primary functionalities. For example, the human skin maintains the ability for sensing the external environment after constant self-repair processes. Inspired by the repeatable self-healing capabilities of living nature, Bao *et al.* designed a new electrically and mechanically self-healing composite as electronic skin.^44^ Xie *et al.* have successfully developed a self-healable and stretchable supercapacitor.^45^ The dynamic PBA–diol ester bonds not only endow our hydrogel with shape memory properties but also with self-healing behaviour.

**Fig. 5:**
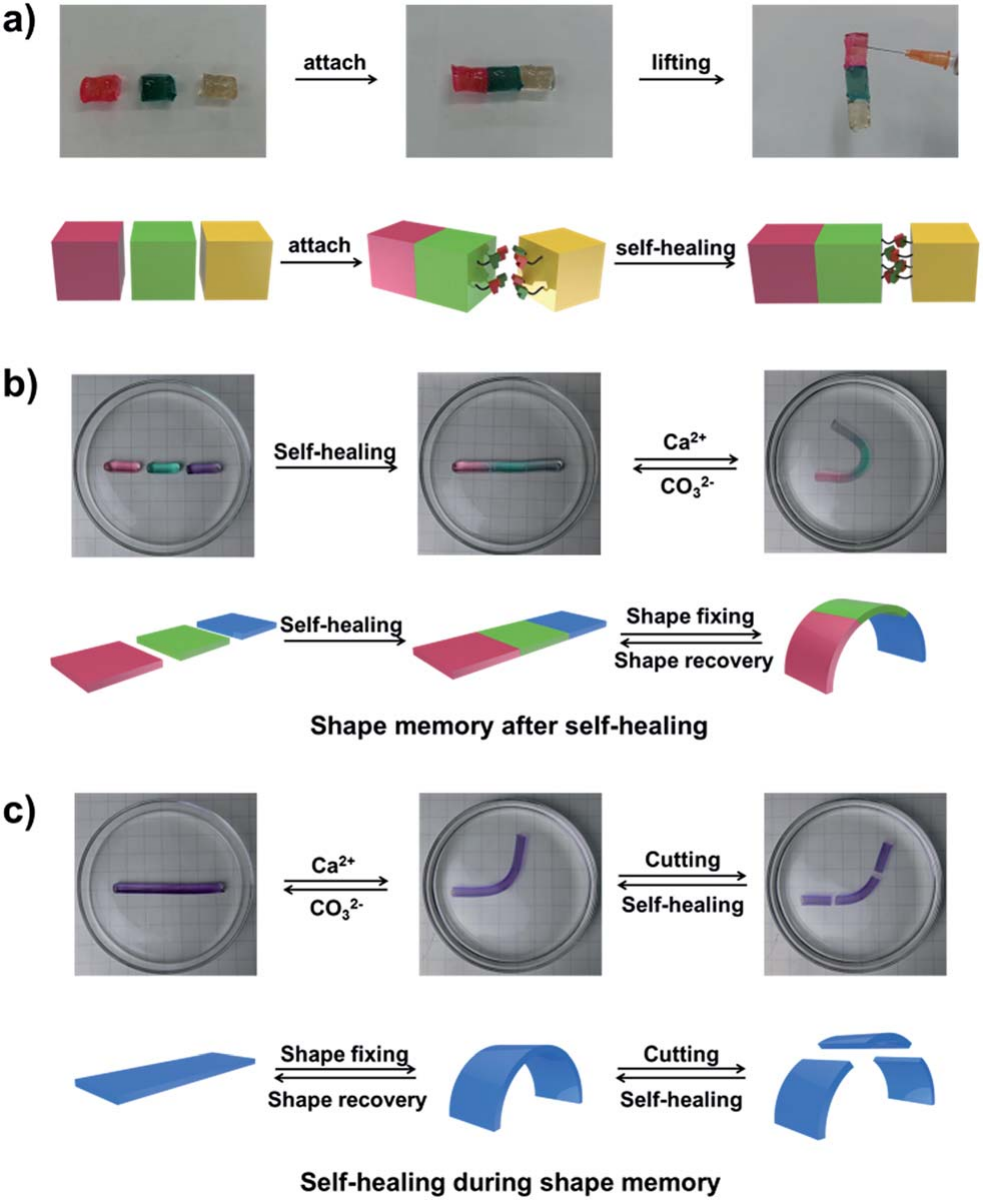
(a) Photographs and mechanism of the self-healing behaviour of the supramolecular hydrogel. Three pieces of hydrogel, where one was dyed with erioglaucine disodium salt (blue dye), and one was dyed with rhodamine B (red dye). The hydrogels are fused together and lift without fracture after fusion. (b) Shape memory after self-healing: a straight hydrogel strip was cut into three parts, and then self-healed at room temperature, the healed hydrogel was bent into a “U” shape and immersed in CaCl_2_ solution to fix the temporary shape, the hydrogel can recover to the original straight shape by extracting Ca^2+^ in K_2_CO_3_ solution. (c) Self-healing during shape memory: a hydrogel strip was first fixed into a temporary shape, and then was cut into three pieces, after self-healing, the healed hydrogel can also recover to the original shape without breakage.

As shown in Fig. 5a, three parts of the supramolecular hydrogel, after cutting from a cylinder sample, are able to self-heal. The manual tensile test and the rheological measurements also confirm the remarkable self-healing ability of the hydrogel (Fig. S13†). The outstanding shape memory and self-healing capabilities of our hydrogel encourage us to explore the combination of the two functionalities. As shown in Fig. 5b, after the self-healing performance of the three pieces of hydrogel, the self-healed hydrogel is bent into a “U” shape and transferred into CaCl_2_ solution to fix the temporary shape *via* the formation of Alg–Ca^2+^ complexation. If Ca^2+^ is erased by CO_3_^2–^, the hydrogel will slowly recover to its original shape without disassociation of the three parts (Fig. S14a†), which indicates the hydrogel still possesses shape memory ability after the self-healing process. The other way around (Fig. 5c), if the hydrogel with a deformed temporary shape stabilized by Alg–Ca^2+^ crosslinks is cut into three pieces, the three pieces will merge into a single one after being brought together, and the self-healed hydrogel could also recover to its original shape (Fig. S14b†). Taking advantage of shape memory as well as self-healing capabilities, the as-prepared hydrogel retains shape memory ability after the self-healing process, and self-healing ability during the shape memory process, which will increase our understanding of the abilities of the living creature and contribute to the creation of novel biomimic materials.

## Conclusions

In conclusion, we have presented the first example of a stretchable supramolecular hydrogel with macro-/microscopic triple shape memory effects. The hydrogel was fabricated with a chemical crosslinked polyacrylamide network and a supramolecular Alg-PBA–PVA network, which renders the hydrogel with good stretchable properties. The non-interfering dynamic PBA–diol ester bonds and the chelation of alginate and Ca^2+^ could be applied as temporary crosslinks to stabilize the deformed shape of the hydrogel, and contribute to the excellent triple shape memory effect at both the macro-scale and micro-scale. Moreover, the hydrogel still possesses shape memory abilities after self-healing, and is capable of self-healing during the shape memory performance, which is comparable with natural biomaterials that can retain primary functionalities after constant self-repair processes. Taking advantage of the double network structure and dual non-interfering supramolecular interactions, we offered a simple and universal approach to construct a mechanical stretchable supramolecular hydrogel with triple shape memory properties, which could broaden the list of shape memory polymers and promote the design and fabrication of novel shape memory systems for a variety of potential biomedical and optical applications.

## Experimental

### Materials

3-Aminophenylboronic acid, sodium alginate (Alg), ammonium persulfate (APS), glycine (≥99.0%), 1-(3-dimethylaminopropyl)-3-ethylcarbodiimide hydrochloride (EDC·HCl), *N*,*N*′-methylene bis(acrylamide) (Bis), rhodamine B (red dye), methyl violet (purple dye) and erioglaucine disodium salt (blue dye) were purchased from Aladdin. Poly(vinyl alcohol) (PVA, 98–99% hydrolyzed, 54–66 viscosity), acrylamide (AAm), calcium chloride (CaCl_2_), sodium hydroxide (NaOH), and potassium carbonate (K_2_CO_3_) were obtained from Sinopharm Chemical Reagent Co., Ltd. APS and AAm were used after recrystallization, and the other chemicals were used without further purification.

### Measurements

^1^H NMR spectra was obtained on a Bruker AVANCE III spectrometer operating at 400 MHz for protons. The tensile and compression tests were conducted on an Instron 5567 Universal Testing System (Instron). The samples for tensile tests were prepared in a dumbbell shape 50 mm in length and 4 mm in width. The samples for compression tests were prepared in a cylinder shape with lengths of 8–10 mm, and diameters of 10 mm. Optical images were taken using a Samsung A7. Scanning electronic microscopy (SEM) measurements were characterized with a Hitachi S4800 microscope. A polarizing microscope BX51-P was used for the observation of microscopic shape memory effects.

### Preparation of Alg-PBA

Alg-PBA was prepared by grafting 3-aminophenylboronic onto an alginate chain in the presence of EDC·HCl according to our previous reports,^30^ the grafting ratio of PBA groups is about 23% (Scheme S1, Fig. S1†).

### Preparation of Alg-PBA–PVA/AAm double network hydrogels

Alg-PBA was mixed with PVA, AAm, APS (initiator) and Bis (crosslinker). The polymerization was carried out at 60 °C for 6 h. After the polymerization step, the hydrogel was transferred into Gly–NaOH solution (pH = 10.6) for 10 min to induce the formation of dynamic PBA–diol ester bonds.
