# Supplementary material for: Stretchable supramolecular hydrogels with triple shape memory effect
Source: Chem Sci. 2016 Jul 7;7(11):6715–20. doi: 10.1039/c6sc02354a (PMC5363791; doi:10.1039/c6sc02354a)
Supplement: Supplementary file 1 [file SC-007-C6SC02354A-s001.pdf]

Supporting information

Stretchable Supramolecular Hydrogels with Triple Shape Memory Effect

Xiaoxia Le,[a] Wei Lu,[a] Jing Zheng,[a] Dingyi Tong,[a] Ning Zhao,[b] Chunxin Ma,[a] He Xiao,[a] Jiawei Zhang,[a] Youju Huang[a] and Tao Chen*[a]*

[a] Ningbo Institute of Material Technology and Engineering, Chinese Academy of Science, Ningbo, 315201, China
[b] Beijing National Laboratory for Molecular Sciences, Laboratory of Polymer Physics and Chemistry, Institute of Chemistry, Chinese Academy of Sciences, Beijing, 100190, China
E-mail: zhangjiawei@nimte.ac.cn, tao.chen@nimte.ac.cn

Preparation of Alg-PBA: Alg-PBA was prepared according to our previous reports (Scheme S1)[1], the content of phenylboronic acid groups in the polymer was calculated to be 23 mol % based on ^1H NMR (D_2O) spectrum (Fig. S1).

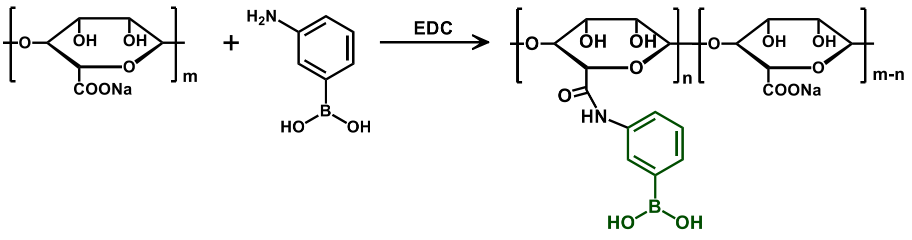

Scheme S1: The synthetic route of Alg-PBA

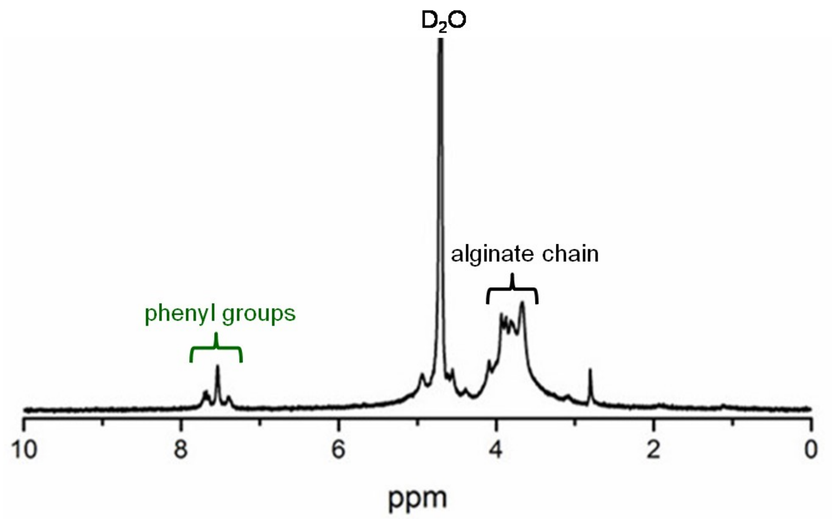

Fig. S1. ^1H NMR(D_2O) spectrum of Alg-PBA.

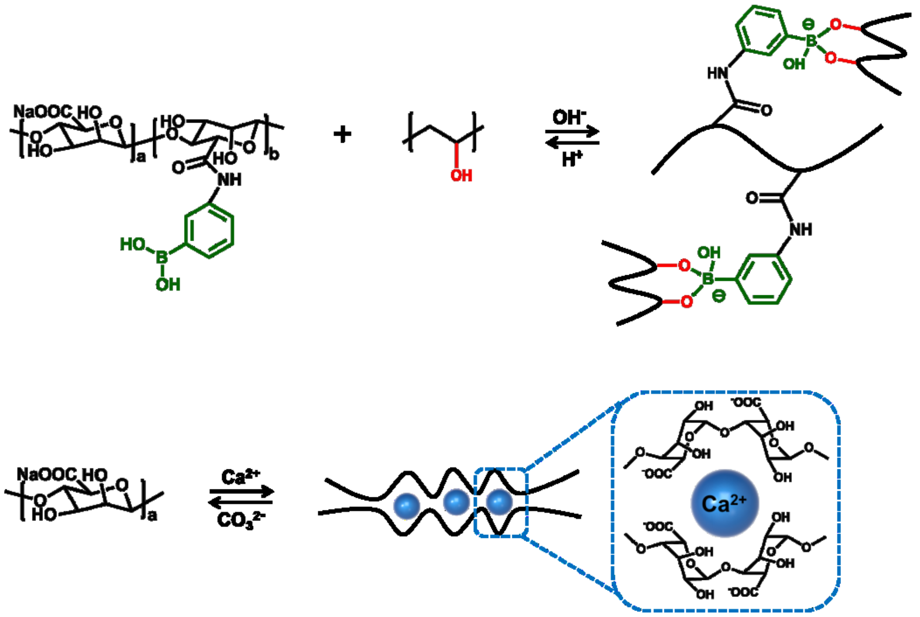

Fig. S2. The reversible PBA-diol ester bonds and Alg-Ca^{2+} crosslinks.

Specific formula of DN hydrogel is shown in Table S1.

Table S1. The formula of A1P3, A1P5 and A1P7 hydrogels.

Sample	Alg-PBA [g]	PVA [g]	AAm [g]	Bis [mg]	APS [mg]	Solid content [%]
A1P3	0.12	0.04	0.49	0.25	4.90	13.04
A1P5	0.12	0.04	0.81	0.41	8.10	18.38
A1P7	0.12	0.04	1.14	0.57	11.40	23.09

Our DN hydrogel contains both supramolecular network based on PBA-diol ester bonds and the chemical crosslinked polyacrylamide network. The morphology of the fabricated hydrogel was investigated by scanning electronic microscopy (SEM), and the supramolecular hydrogel shows typical porous structure (Fig. S3), indicating the two networks are well mixed.

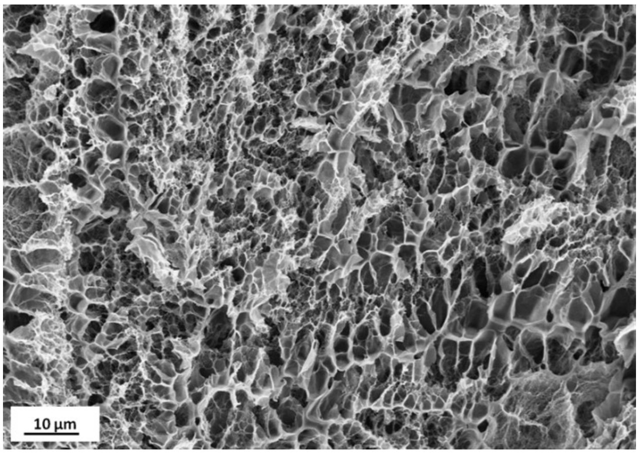

Fig. S3. SEM images of freeze-dried A1P5 hydrogel.

A strip with 10 mm in width, 20 mm in length (ignore the part in the clips) and 2 mm in thickness was prepared for manual stretching. As shown in Fig. S4a, the A1P5 hydrogel can be stretched to more than 12 times of its original length and recover to its initial state, and it is also tough enough to withstand high deformation in compression without obvious damage (Fig. S4b).

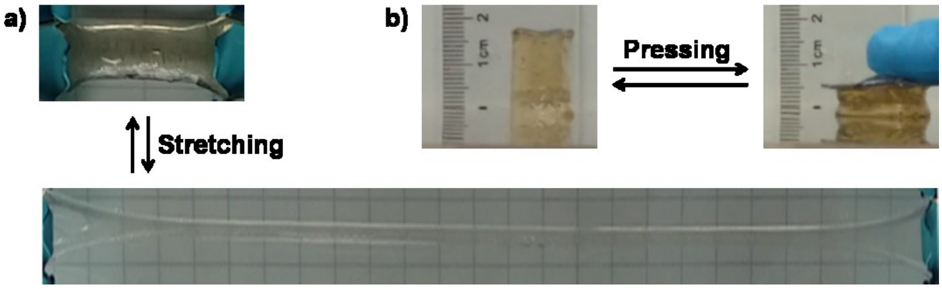

Fig. S4. Stretching (a) and compressing (b) of the A1P5 hydrogels.

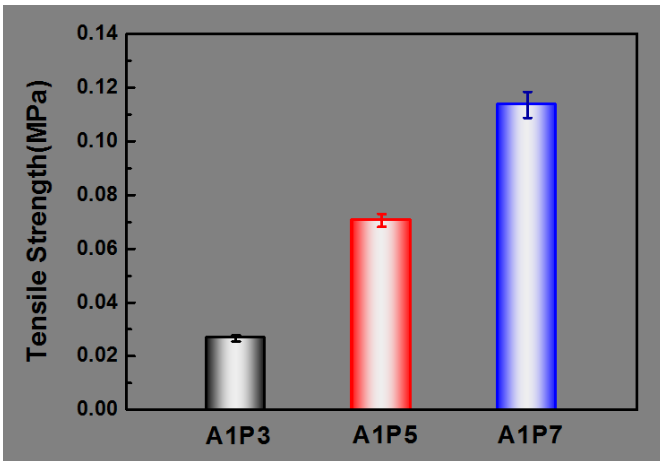

Fig. S5. The bar graph of tensile stress of DN hydrogels with different network ratio.

The compressive test of A1P3, A1P5 and A1P7 hydrogels were measured at a compression rate of 10% original height/min and the final compressive strain is 98%. As shown in Fig. S6, neither A1P3 nor A1P5 hydrogels can recover to their original state, only A1P5 hydrogel shows better compression performance.

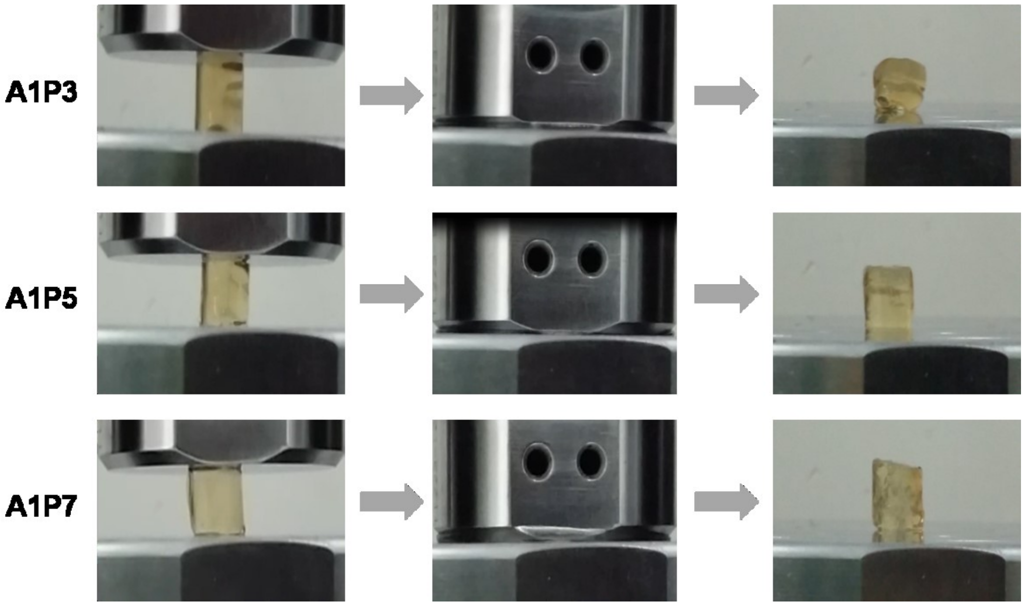

Fig. S6. The photographs of compression test of A1P3, A1P5 and A1P7 hydrogels.

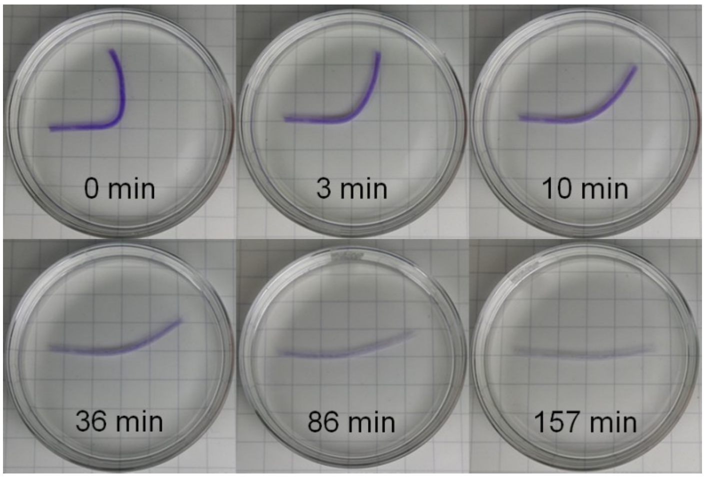

Fig. S7. The recovery procedure of A1P5 hydrogel in 0.1 M K_2CO_3 solution.

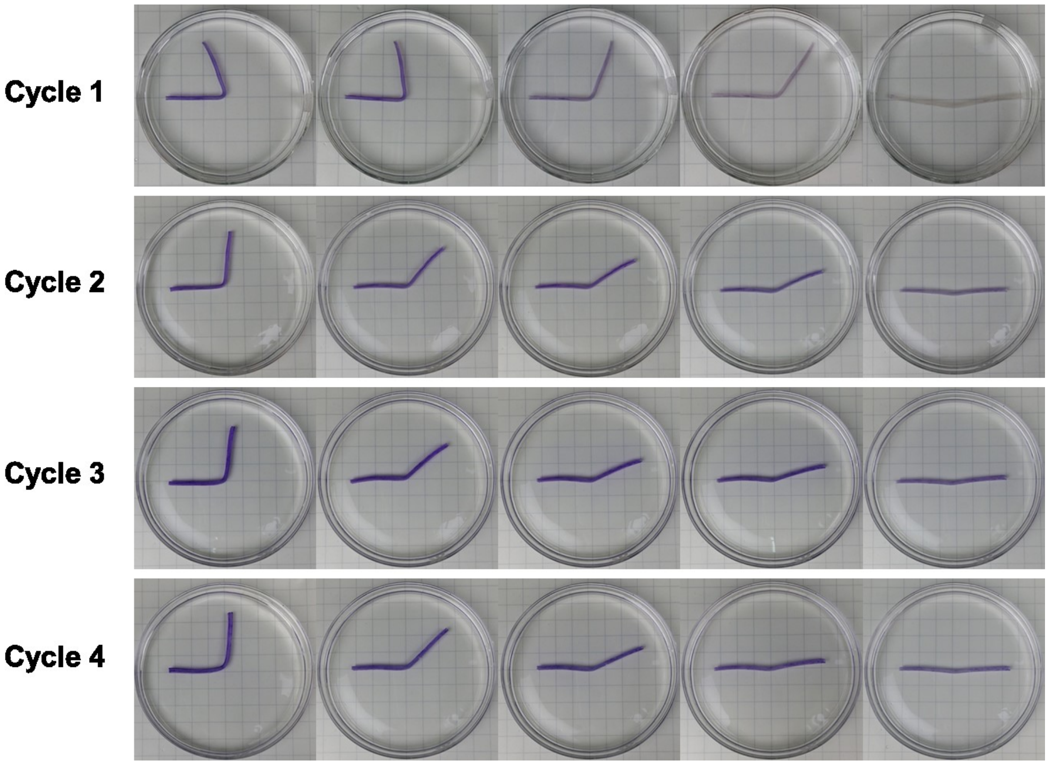

Fig. S8: Cycled shape memory behavior of A1P5 hydrogel based on Alg-Ca^{2+} interactions. K_2CO_3 solution (0.1 M) was used for shape recovery. For better vision, the hydrogel was dyed again after each cycle.

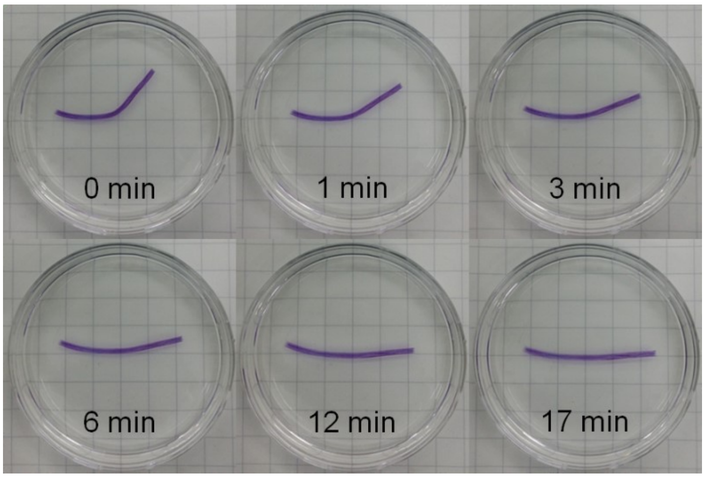

Fig. S9. The recovery procedure of A1P5 hydrogel in Gly solution (pH=6).

The recover process was taken first in Gly aqueous solution to break PBA-diol ester bonds. After washing in deionized water, the hydrogel was then put into K_2CO_3 aqueous solution to remove Ca^{2+}, and the hydrogel can finally recover to its original shape, as shown in Fig. S10.

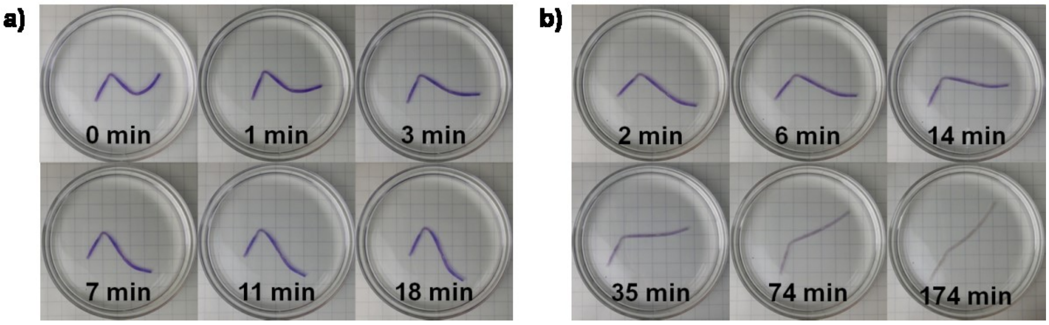

Fig. S10. Programmed shape recovery process. Specific recover process of programmed shape memory hydrogel a) recovery in Gly solution (pH = 6), b) recovery in K_2CO_3 solution (0.1M).

The stretching shape memory and releasing shape recovery were shown in Fig. S11. Firstly, hydrogel was cut into strips with 10 mm in width, 30 mm in length and 1 mm in thickness. 2) A hydrogel strip (effective length 2 cm) was stretched to 4 times of original length (8 cm), and immersed in $CaCl_2$ solution for 1 min. The actual memorized length was 2 times of the original length (4 cm). 3) Then the stretched hydrogel was extended to 8 cm, and kept in Gly-NaOH solution (pH = 10.6). As a result, the finally length of the hydrogel was 3 times of original length (6 cm). 4) Finally, the hydrogel was stepwise put into Gly and K_2CO_3 solution for shape recovery. Though the whole recover ratio is as high as 100%, the shape fixing ratio is only 75% after two memorize steps, which indicates that the double network structure improves the mechanical strength, as well as endows hydrogel with stretch resistance.

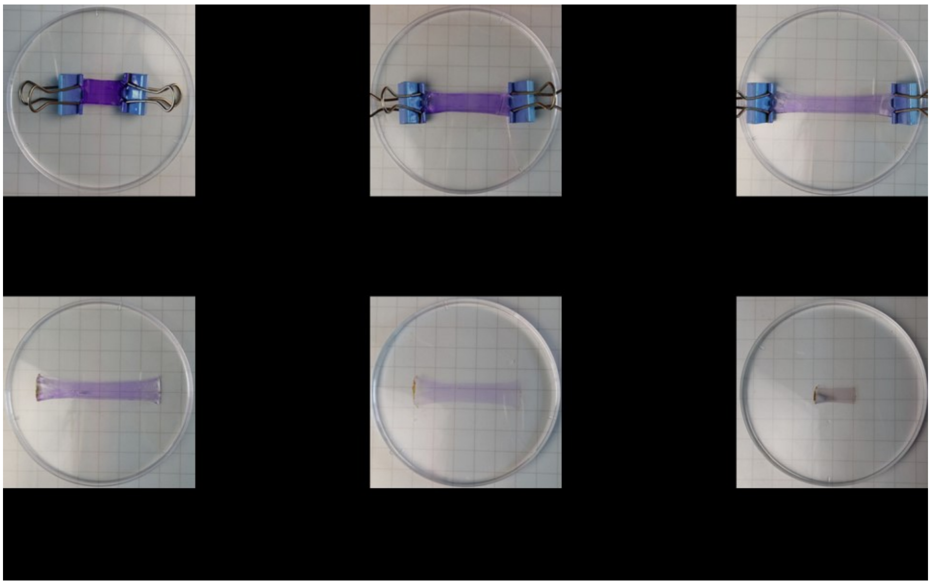

Fig. S11. The images of stretching shape memory and releasing shape recovery.

The shape recovery of the micro-patterned surface is shown in Fig. 12, after immersing into Gly aqueous solution to break the PBA-diol ester bonds, the hydrogel with line patterns in the shape recovery process is almost identical with the middle images of Fig. 4b, which suggests the shape recovery ratio is almost 100 %.

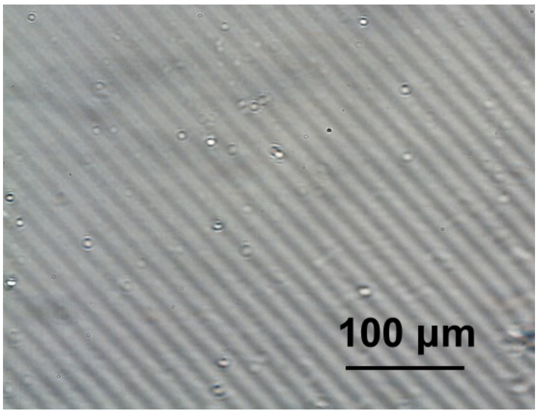

Fig. S12. The image of hydrogel with line patterns in shape recovery process.

As shown in Fig. S13a, the A1P5 hydrogel with diameter 6 mm was cut into two pieces and brought together immediately. After 24 h in room temperature, the joint of the healed hydrogel is strong enough to be stretched. The hydrogel needs a long time to recover the mechanical properties, which indicates the high content of chemical crosslinked PAAm will hinder the self-healing process. Figure S13b shows the rheological results. When a large-amplitude oscillatory (γ = 600%, frequency = 1.0 Hz) was applied, under a 3% strain, G′ is much larger than G″, implying the formation of self-standing hydrogel. However, if a large-amplitude oscillatory (γ = 600%, Frequency = 1.0 Hz) was applied, the G′ decreases from 2500 Pa to 658 Pa while the G″ increases from 415 Pa to about 720 Pa, which suggests the hydrogel collapses to quasi-liquid state. G′ and G″ recover quickly to initial values by decreasing the amplitude (γ =3%, frequency = 1.0 Hz), this recovery behavior can be repeated for at least three cycles. In addition, G′ becomes lower and lower after each circle, which may be caused by the damage of chemical crosslinked PAAm. Both the manual tensile test and the rheological measurement suggest our hydrogel has self-healing ability.

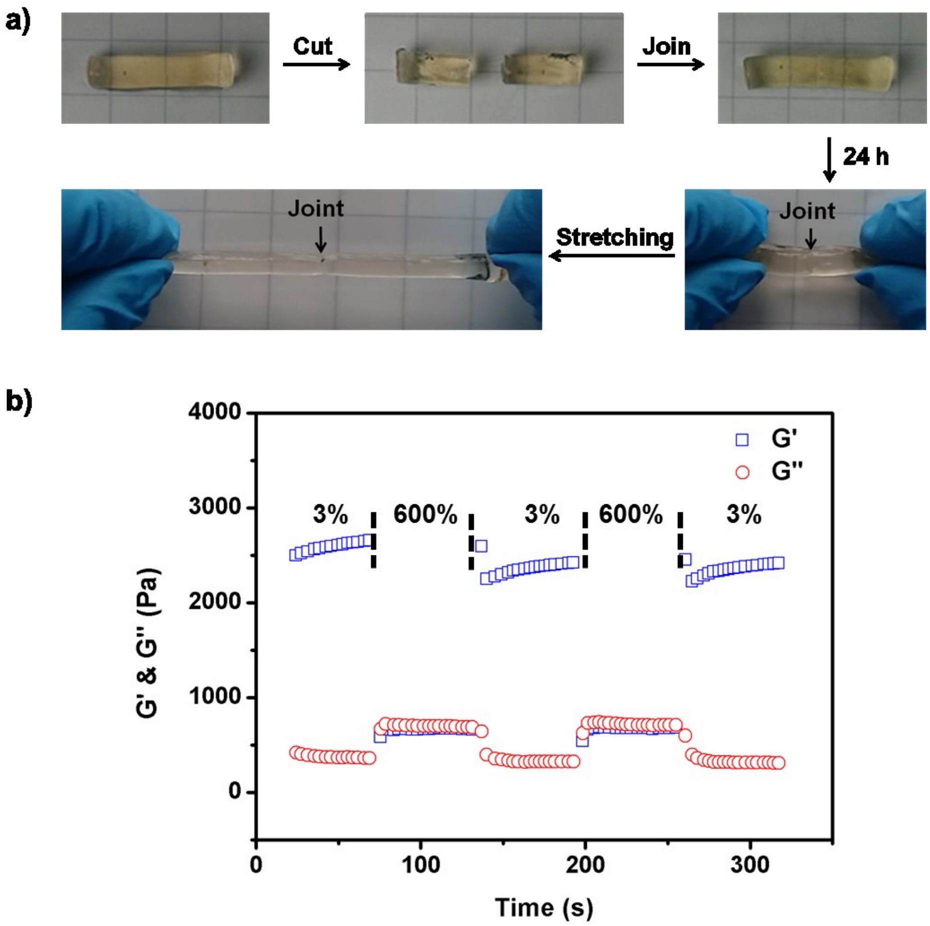

Fig. S13. a) Pictures of the self-healing process and stretching of the healed hydrogel. b) Storage modulus (G′) and loss modulus (G″) of the as-prepared hydrogel in continuous step strain measurements.

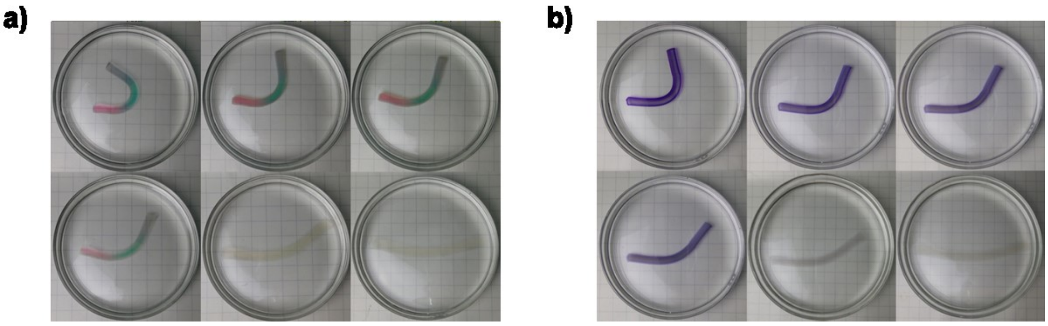

Fig. S14. a) Specific recover process of the hydrogel which was self-healed before shape fixing; b) Specific recover process of the hydrogel which was self-healed after shape-fixing.

Supplementary Movie S1, Movie S2

Movie S1 shows the manual stretching of A1P5 hydrogel, Movie S2 shows the stretching of A1P5 hydrogel at an extension rate of 50 mm/min.

Supplementary Movie S3

Movie S3 shows the compressing of A1P3, A1P5 and A1P7 hydrogel.

Reference

1. H. Meng, P. Xiao, J. C. Gu, X. F. Wen, J. Xu, C. Z. Zhao, J. W. Zhang, T. Chen, *Chem. Commun.* 2014, **50**, 12277-12280.
